# Watching the Insane by Reflecting Shafts

**Published:** 1890-05-10

**Authors:** 


### Watching the Insane by Reflecting Shafts.

It is very generally admitted that our English County Asylums are liberally supplied with every appliance that can make the life of the lunatic comfortable or tend to induce recovery, and it is probable that the American State Hospitals for the insane are not far behind us in these things. But there would seem to be a feeling here, and in the States, that the direct supervision of the patients is not quite what it ought to be, and various suggestions have been made from time to time. One of the latest, which is, however, now an accomplished fact, is that of Dr. Chase, of the Norristown Asylum in the United States. Dr. Chase has invented a series of shafts which connect the ground floors with the first floors of his Asylum. These shafts open into small rooms called Inspection rooms, and here sits an inspector who, by an arrangement of mirrors placed at either end of the shaft can see what is going on in the wards, something like the style so common in Dutch towns whereby a little mirror is fixed outside the window, and by placing it at a certain angle, everything which goes on at the end of the street is seen in the room. Dr. Chase points out that the system equally protects the patients against the rough usage of an attendant and the attendant against the false charge of a patient. The shaft acts also as a sort of speaking tube, so that any unusual noise can be heard. One would fancy it would also act as a ventilating medium, and draw the foul air into the inspector's room. As to the utility of the system, everything would depend on the vigilance of the inspector. He would be something like a pointsman on a railway, and we should fear that the duty of looking at reflecting panoramas would soon get irksome beyond endurance. Dr. Chase, too, admits that "in the system an occasional blow, kick, or other instantaneous act of violence might be visited on a patient; but I am positive that no systematic course of abuse can now occur." Now we think that if the system fail to secure protection against instantaneous acts that it would practically fail altogether, as far as English county asylums are concerned, because we do not think that anything like systematic ill-treatment takes place in any of our asylums. With the "points" of Dr. Chase's system, as stated in the newspaper report, from which we have drawn the above particulars, it is almost superfluous to say that we are in full accord. These points are: 1st, properly remunerating faithful service by a system of increase of wages, so as to encourage good attendants to remain; 2nd, to institute a system of inspection in wards by employing thoroughly reliable men at suitable salaries, who shall be excused from hard work; 3rd, to establish a system of training of the attendants for the general improvement of the service by the inspectors and lectures by the physicians; 4th, to make improvements from time to time in the comfort of attendants, to render their lives healthful and cheerful. No. 1, No. 3, and No. 4 are universally recognized and acted on to a greater or less extent in our English asylums, and the second has certainly been adopted in more than one asylum. If it were invariable, one great step in advance would have been made, and the system of supervision of unreflecting attendants by reflecting glasses would be wholly unnecessary. The difficulties which stop the way are, 1st, the expense, which must always carry weight in a rate-supported institution; 2nd, the uncertainty of obtaining really suitable people; 3rd, the difficulty in most asylums of providing accommodation and attendance for these sub-officers, as they would have to be called.

